# Speech of the Governor of the Commonwealth

**Published:** 1865-07

**Authors:** 


At this point, His Excellency the Governor of the Commonwealth, preceded by the Sergeant-at-Arms, and accompanied by Assistant-Surgeon-General Hooker, entered the hall, and was received by the Association, the members rising in their seats. His Excellency was introduced by the President, and addressed the Association as follows:—

*Mr. President, and Gentlemen of the Convention:*—I did not presume to enter the hall with the intention of interrupting the proceedings of the body which has met beneath the arches of the Hall of the House of Representatives, but for the purpose, in the first place, of testifying by my presence and my voice, and to respectfully acknowledge the honor conferred upon me by the Convention, in the invitation to attend and to address this meeting on the morning of its first convening. Having been prevented by illness from doing so, I felt it due to myself and the body assembled, to avail myself of the earliest practical moment of passing a short time in the presence of this large assembly of the most learned and wisest of the medical profession; but more, I must say, I felt it to be due to the Commonwealth, one of whose official representatives I am, to testify in praise, and with emphasis also, to the interest which it attaches to such meetings of gentlemen of the learned profession of medicine. The interest it attaches to it, not only from its relation to the cause of science, but in relation to the cause of popular progress in many of the departments of human learning. The interest also which it attaches to the medical profession in its relation to the patriotism of the people, since that profession represents the body of men whose devotion, whose energy, whose skill, whose ability, and whose usefulness have not been surpassed, during these last four years of Southern war, by any similar number of citizens of the United States. [Applause.] And I trust, Mr. President, that this meeting in the City of Boston will be but the introduction of your Association to the city, and that the capital of the Commonwealth may witness many returns of a similar reunion. And I hope that wherever your honorable body may meet hereafter, it will receive the same cordial welcome of an intelligent people capable of appreciating learning, capable of understanding adequately and valuing the contributions of such men, alike to the health and morals, and to the sentiments of the people. For I believe, sir, that reasonable, progressive, and philosophic views, in regard to the treatment of disease, or deranged health in all the forms of altered life, are both useful in respect to that which is perishable in our mortal bodies, and in respect to that which is eternal in the immortal mind. [Cheers.] I am happy to learn, Mr. President, that the meeting of the Convention has been attended on the present occasion with so many circumstances of interest and value, and cordially thanking you for this opportunity of addressing the Convention, and wishing the body themselves future happiness and prosperity, for many years to come, I have the honor to take my leave. [Cheers.]

At the conclusion of his address His Excellency retired, and the Association resumed its business.

